# Supplementary material for: The manganese transporter SLC39A8 links alkaline ceramidase 1 to inflammatory bowel disease
Source: Nat Commun. 2024 Jun 5;15:4775. doi: 10.1038/s41467-024-49049-8 (PMC11153611; doi:10.1038/s41467-024-49049-8)
Supplement: Supplementary file 1 — Supplementary information [file 41467_2024_49049_MOESM1_ESM.pdf]

Supplementary Information for

The manganese transporter SLC39A8 links alkaline ceramidase 1 to inflammatory bowel disease

Eun-Kyung Choi[1], Thekkelnaycke M. Rajendiran[2,3], Tanu Soni[3], Jin-Ho Park[1], Luisa Aring[1], Chithra K. Muraleedharan[2], Vicky Garcia-Hernandez[2], Nobuhiko Kamada[2,4], Linda C Samuelson[4,5], Asma Nusrat[2], Shigeki Iwase[6], and Young Ah Seo[1*]

[1]Department of Nutritional Sciences, University of Michigan School of Public Health, Ann Arbor, Michigan, USA.
[2]Department of Pathology, University of Michigan Medical School, Ann Arbor, Michigan, USA.
[3]Michigan Regional Comprehensive Metabolomics Resource Core, University of Michigan Medical School, Ann Arbor, Michigan, USA.
[4]Division of Gastroenterology and Hepatology, Department of Internal Medicine, University of Michigan Medical School, Ann Arbor, Michigan, USA.
[5]Department of Molecular and Integrative Physiology, University of Michigan Medical School, Ann Arbor, Michigan, USA.
[6]Department of Human Genetics, University of Michigan Medical School, Ann Arbor, Michigan, USA.

* Corresponding author. E-mail: youngseo@umich.edu

Supplementary Figure 1

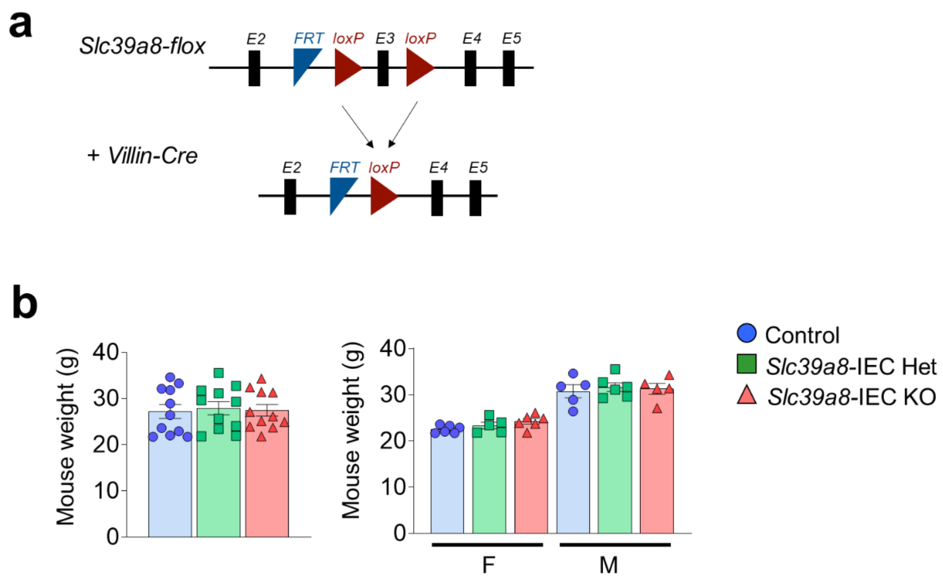

Supplementary Fig. 1. Generation of *Slc39a8* IEC-KO mice. (**a**) Schematic representation of mice with deletion of *Slc39a8* in the intestine. (**b**) Mouse body weight in 20-week-old control and *Slc39a8*-IEC KO mice. [control : *n* = 11, *n* = 5 male (M), *n* = 6 female (F); *Slc39a8*-IEC Het : *n* = 11, *n* = 5 M, *n* = 6 F *Slc39a8*-IEC KO : *n* = 11, *n* = 5 M, *n* = 6 F]. Data are presented as individual values and represent the mean ± SEM. The *p*-values were determined by one-way ANOVA with Bonferroni's multiple comparisons test for **b**. Source data are provided as a Source Data file.

Supplementary Figure 2

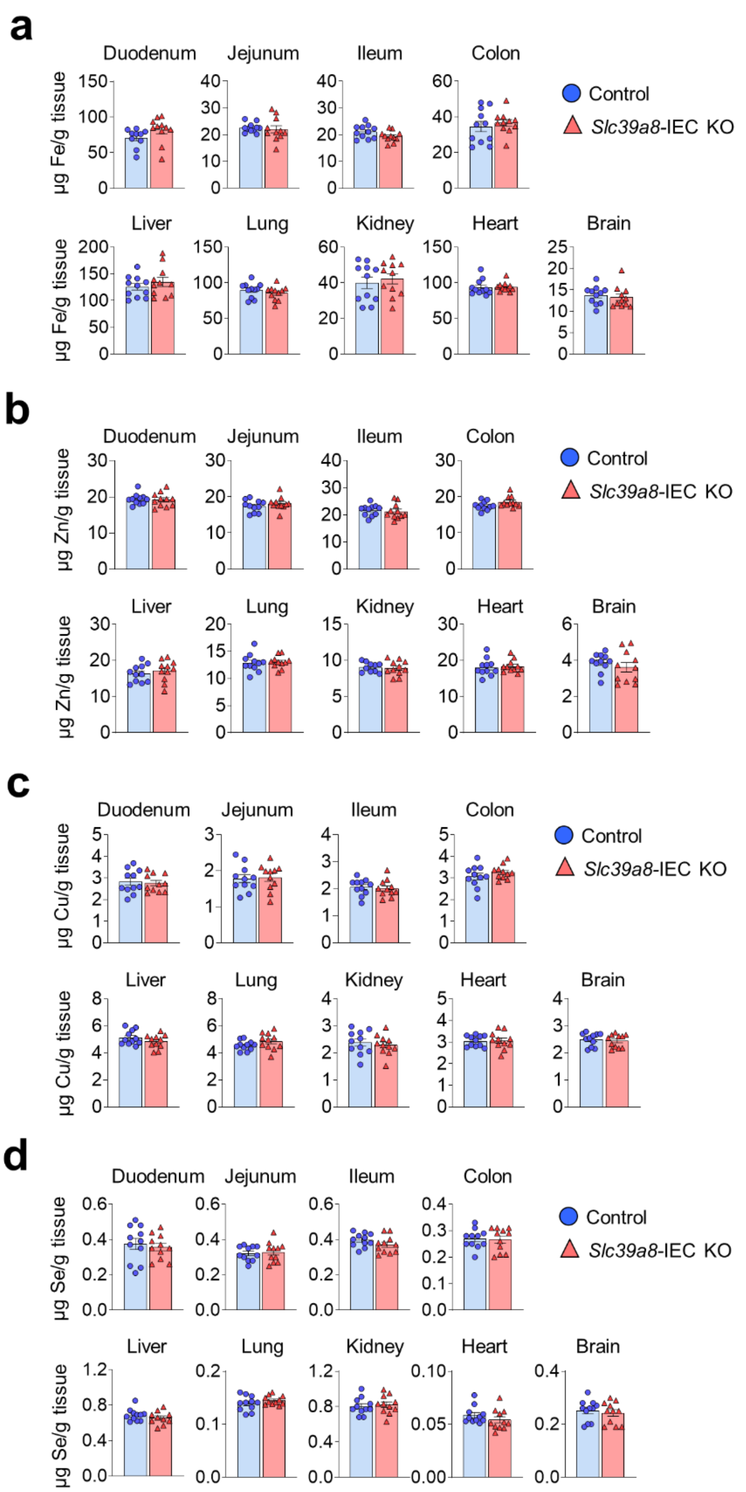

Supplementary Fig. 2. Iron (Fe), zinc (Zn), copper (Cu), and selenium (Se) levels in control and *Slc39a8*-IEC KO mouse models. (**a-d**) ICP-MS analysis of (**a**) Fe, (**b**) Zn, (**c**) Cu, and (**d**) Se levels in 20-week-old female and male control and *Slc39a8*-IEC KO mice. [control : *n* = 11, *n* = 5 male (M), *n* = 6 female (F); *Slc39a8*-IEC Het : *n* = 11, *n* = 5 M, *n* = 6 F *Slc39a8*-IEC KO : *n* = 11, *n* = 5 M, *n* = 6 F]. Data are presented as individual values and represent the mean ± SEM. The *p*-values were determined by unpaired two-tailed Student's *t*-test for **a, b,** and **c**. Source data are provided as a Source Data file.

Supplementary Figure 3

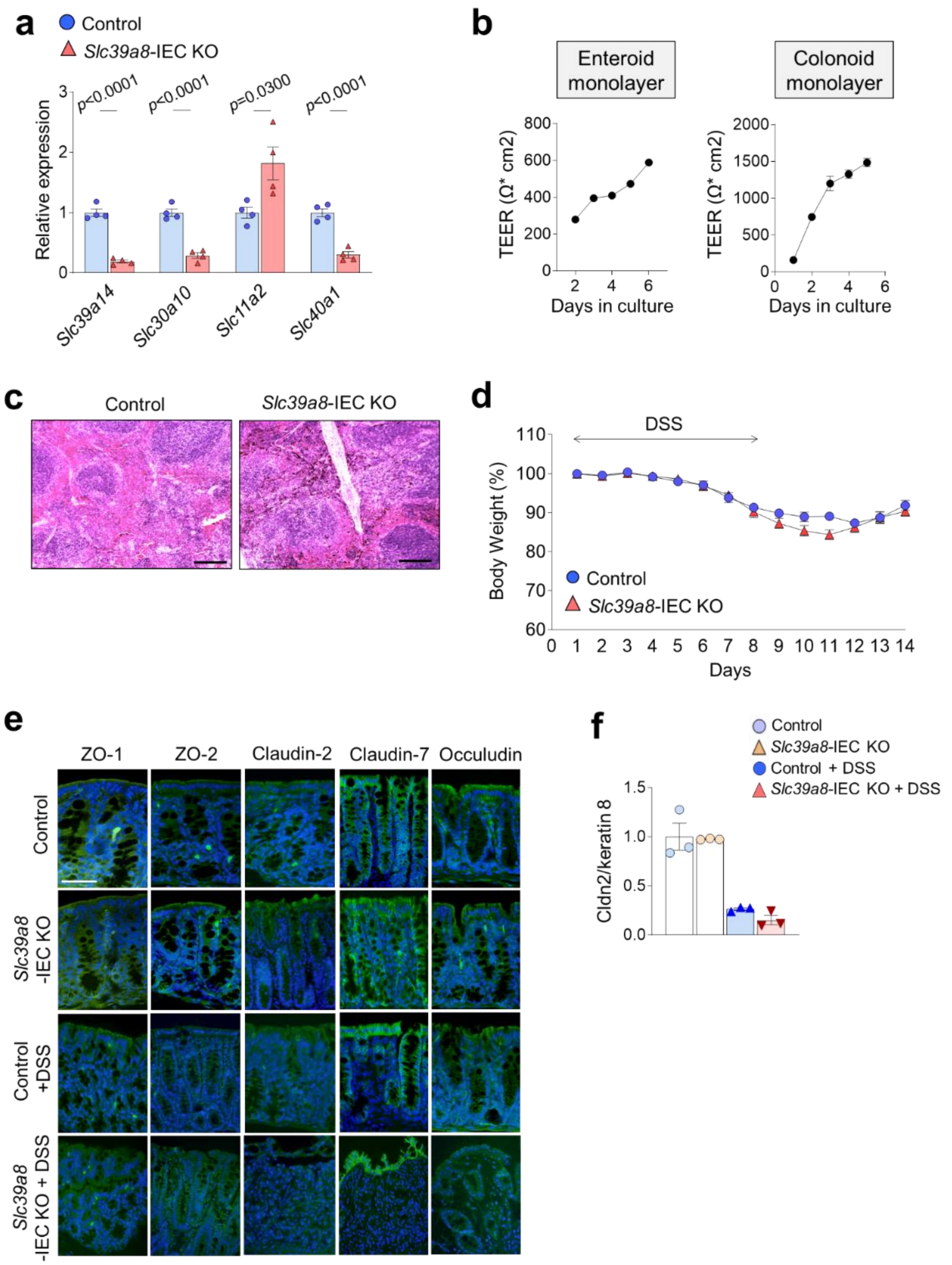

Supplementary Fig. 3. Transporter RNA levels in *Slc39a8*-IEC KO mouse-derived intestinal organoid monolayer culture and dextran sodium sulfate (DSS)-induced colitis in *Slc39a8*-IEC KO mice. (**a**) Transporter RNA levels were measured in enteroid monolayers derived from control and *Slc39a8*-IEC KO mice. (**b**) Intestinal transepithelial electrical resistance (TEER) was measured in both enteroid and colonoid monolayers derived from control mice (*n* = 12 per group). (**c**) H&E staining of the spleen on day 13 after DSS treatment in control and *Slc39a8*-IEC KO mice. Scale bars: 100 µm. (**d**) Female *Slc39a8*-IEC KO mice display lower sensitivity to DSS in terms of DSS-induced colitis. Changes in body weight (percentage of original body weight) over time (days) in 8-week-old female control and *Slc39a8*-IEC KO mice following DSS treatment (*n* = 5 per group). (**e**) Immunofluorescence staining of tight junction proteins. (**f**) Quantification of relative Cldn2 protein expression after normalization with keratin 8 (*n* = 3 biologically idependent samples). Data are presented as individual values and represent the mean ± SEM. The *p*-values were determined by unpaired two-tailed Student's *t*-test for **a,** two-way ANOVA with Bonferroni's multiple comparisons test for **d,** and one-way ANOVA with Bonferroni's multiple comparisons test for **f**. Source data are provided as a Source Data file.

Supplementary Figure 4

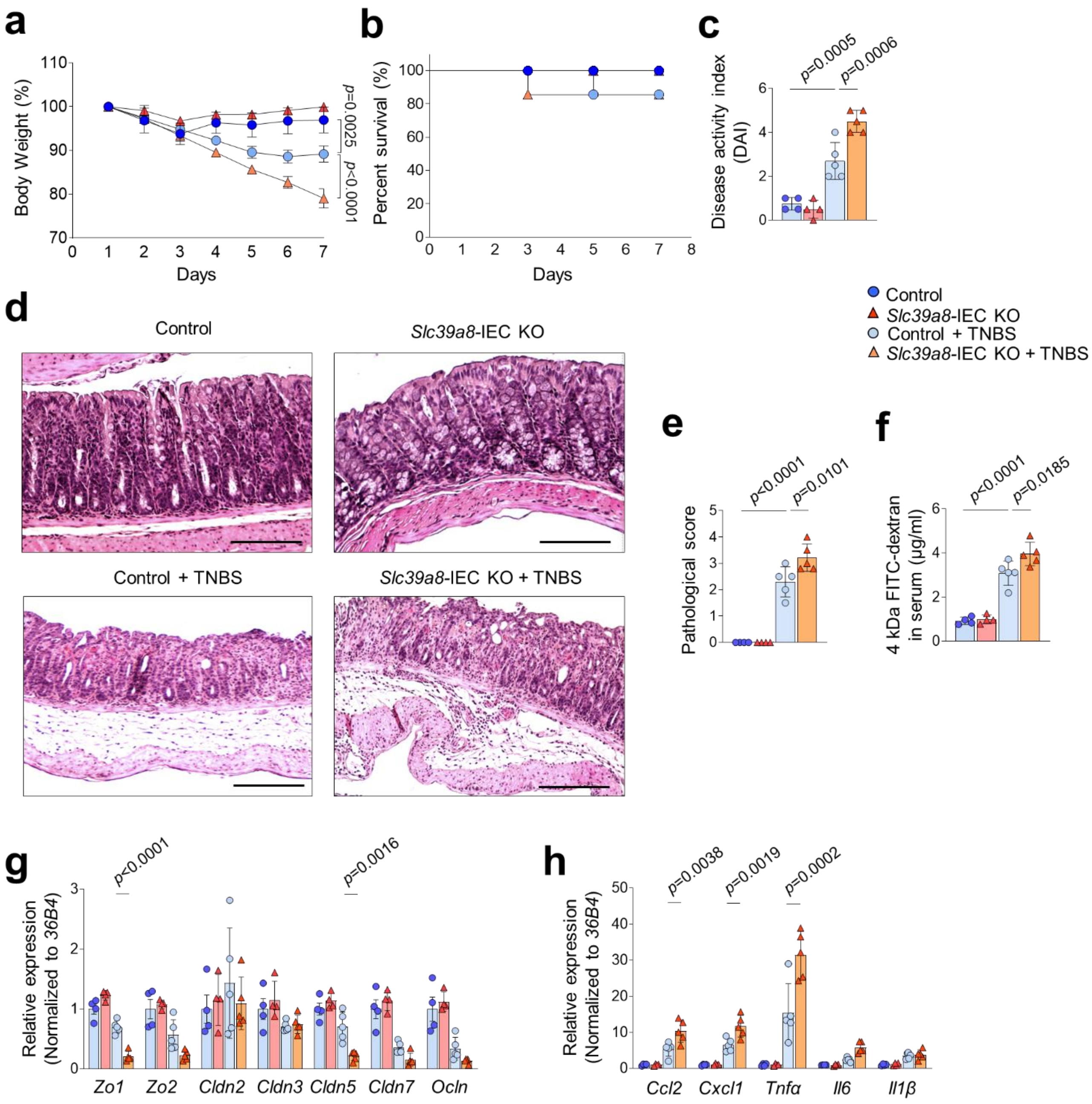

Supplemental Fig. 4. Loss of *Slc39a8* in IEC exacerbates 2,4,6-trinitrobenzene sulfonic acid (TNBS)-induced colitis. Six-week-old male control and *Slc39a8*-IEC KO mice were injected with 50% ethanol (Vehicle) or TNBS at day 1 to induce acute colitis for disease analysis, and mice were sacrificed after 7 days. (**a**) Changes in body weight (percentage of original body weight) over time (days) (Control, n = 4; Slc39a8-IEC KO, n = 4; Control + TNBS, n = 5; Slc39a8-IEC KO + TNBS, n = 5 per group). (**b**) Percent survival (%). (**c**) Disease activity index (DAI) (Control, n = 4; Slc39a8-IEC KO, n = 4; Control + TNBS, n = 5; Slc39a8-IEC KO + TNBS, n = 5 per group). (**d**) Hematoxylin/eosin (H&E) staining of colons and (**e**) pathological scores (Control, n = 4; Slc39a8-IEC KO, n = 4; Control + TNBS, n = 5; Slc39a8-IEC KO + TNBS, n = 5 per group). Scale bars: 100 μm. (**f**) Measurement of 4 kDa FITC-dextran in serum (Control, n = 4; Slc39a8-IEC KO, n = 4; Control + TNBS, n = 5; Slc39a8-IEC KO + TNBS, n = 5 per group). (**g** and **h**) qPCR quantification of (**G**) tight junction and (**H**) proinflammatory cytokines and chemokines in colon mucosa (Control, n = 4; Slc39a8-IEC KO, n = 4; Control + TNBS, n = 5; Slc39a8-IEC KO + TNBS, n = 5 per group). Data are presented as individual values and represent the mean ± SEM. The p-values were determined by two-way ANOVA with Bonferroni's multiple comparisons test for **a,** log-rank test for **b,** and one-way ANOVA with Bonferroni's multiple comparisons test for **c, e, f, g,** and **h**. Source data are provided as a Source Data file.

Supplementary Figure 5

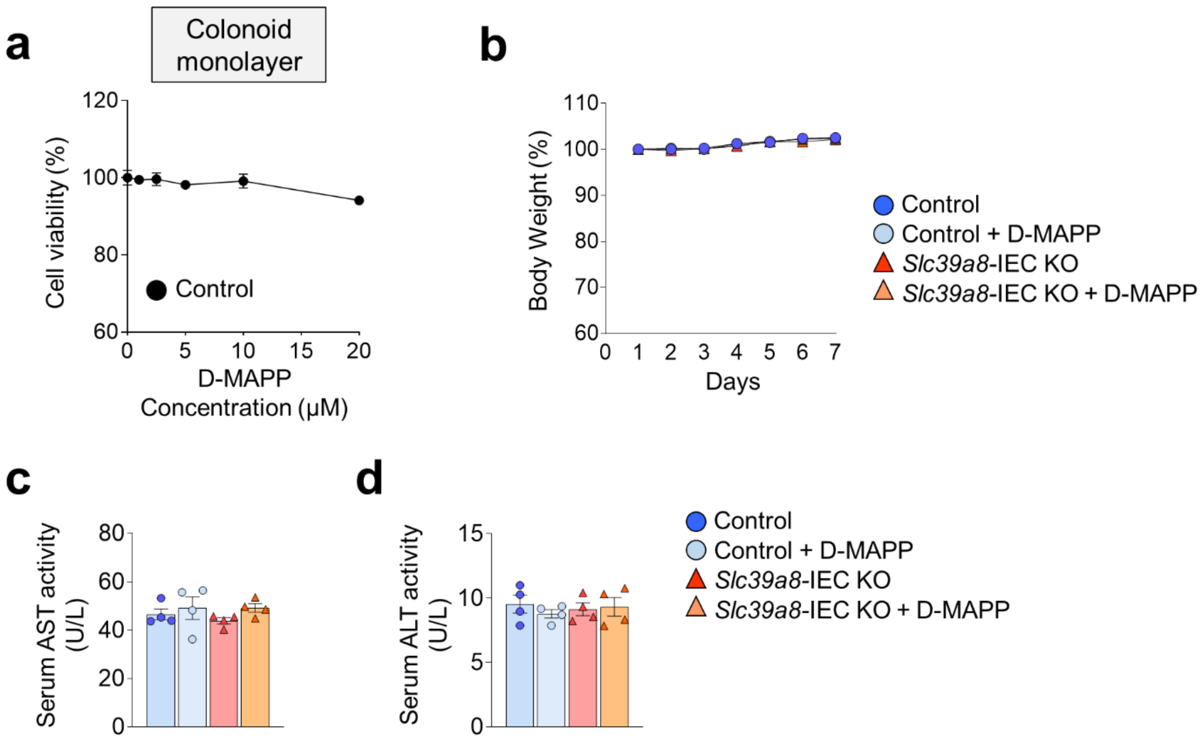

Supplementary Fig. 5. Evaluation of (1S,2R)-d-erythro-2-(N-myristoylamino)-1-phenyl-1-propanol (D-*e*-MAPP) toxicity *in vitro* and *in vivo*. (**a**) Cell viability of intestinal organoid monolayer cultures with D-*e*-MAPP at the indicated doses. (**b**) Changes in body weight (percentage of original body weight) over time (days) during pretreatment with D-*e*-MAPP (n = 4 per group). (**c, d**) Serum AST and ALT levels after 7 days of pretreatment with D-*e*-MAPP (n = 4 per group). Data are presented as individual values and represent the mean ± SEM. The p-values were determined by two-way ANOVA with Bonferroni's multiple comparisons test for **b,** and one-way ANOVA with Bonferroni's multiple comparisons test for **c** and **d**. Source data are provided as a Source Data file.

Supplementary Figure 6

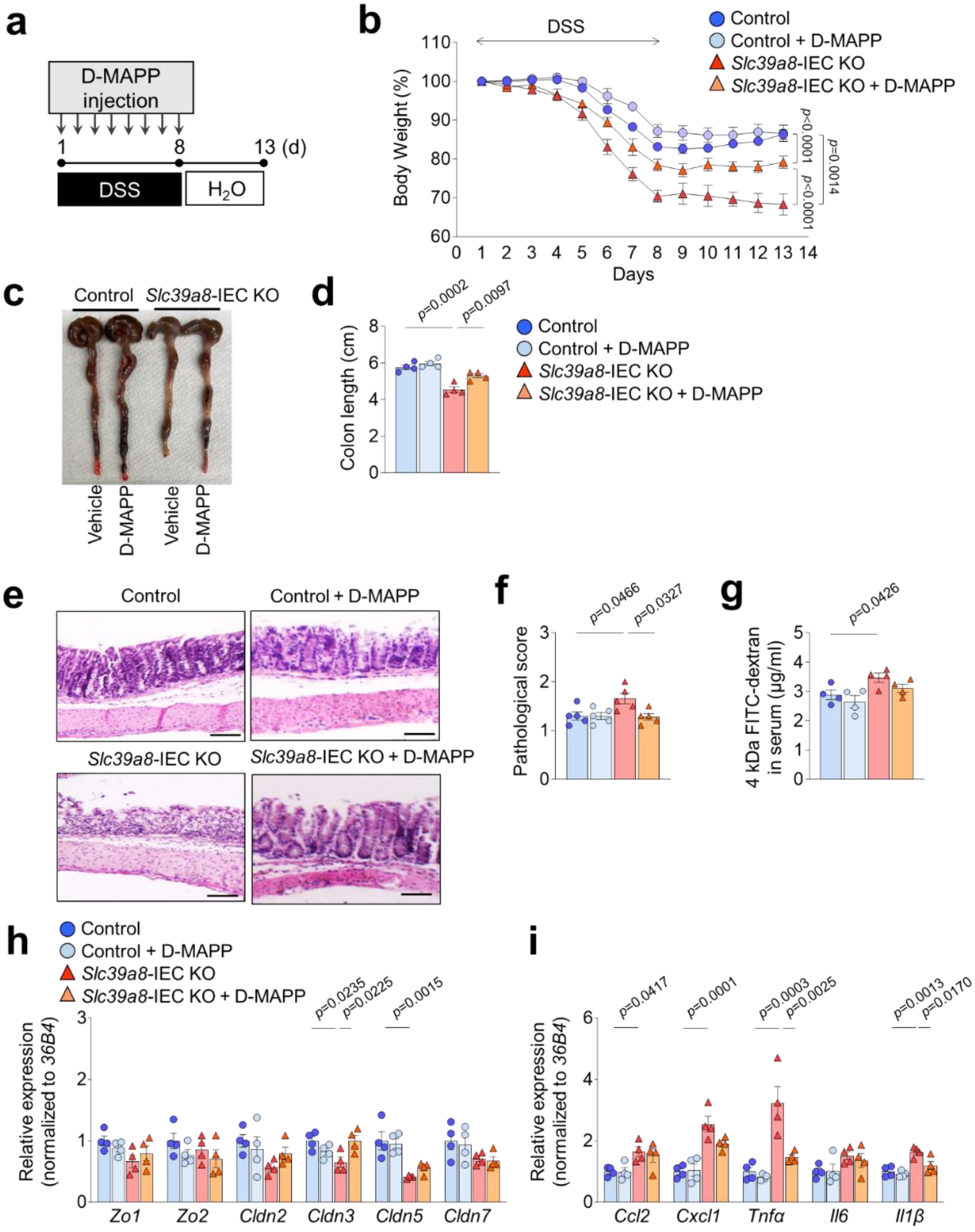

Supplementary Fig. 6. Treatment with the ACER1 inhibitor (1S,2R)-d-erythro-2-(N-myristoylamino)-1-phenyl-1-propanol (D-*e*-MAPP) mitigates colitis *in Slc39a8*-IEC KO mice. (**a**) Schematic of the ACER1 inhibitor D-*e*-MAPP treatment in a dextran sodium sulfate (DSS)-induced colitis model. Mice were treated with or without the ACER1 inhibitor D-*e*-MAPP (10 nmol/g body weight) at 24 h intervals ending at day 8. For colitis induction, mice were provided with drinking water containing 3% (w/v) DSS for 8 days (inflammatory phase). The mice were then provided with regular drinking water for 5 days (recovery phase). (**b**) Changes in body weight (percentage of original body weight) over time (days) in 8-week-old male mice following DSS treatment (*n* = 4 per group). (**c**) Gross morphology of the large intestine. (**d**) Colon length on day 13 after DSS treatment (*n* = 4 per group). (**e**) Hematoxylin/eosin (H&E) staining of colons and (**f**) pathological scores (*n* = 5 per group). Scale bars: 100 μm. (**g**) 4 kDa FITC-dextran in serum (*n* = 4 per group). (**h** and **i**) qPCR quantification of (**h**) tight junction and (**i**) proinflammatory cytokines and chemokines in colon mucosa (*n* = 4 per group). Data are presented as individual values and represent the mean ± SEM. The *p*-values were determined by two-way ANOVA with Bonferroni's multiple comparisons test for **b,** and one-way ANOVA with Bonferroni's multiple comparisons test for **d, f, g, h,** and **i**. Source data are provided as a Source Data file.

Supplementary Figure 7

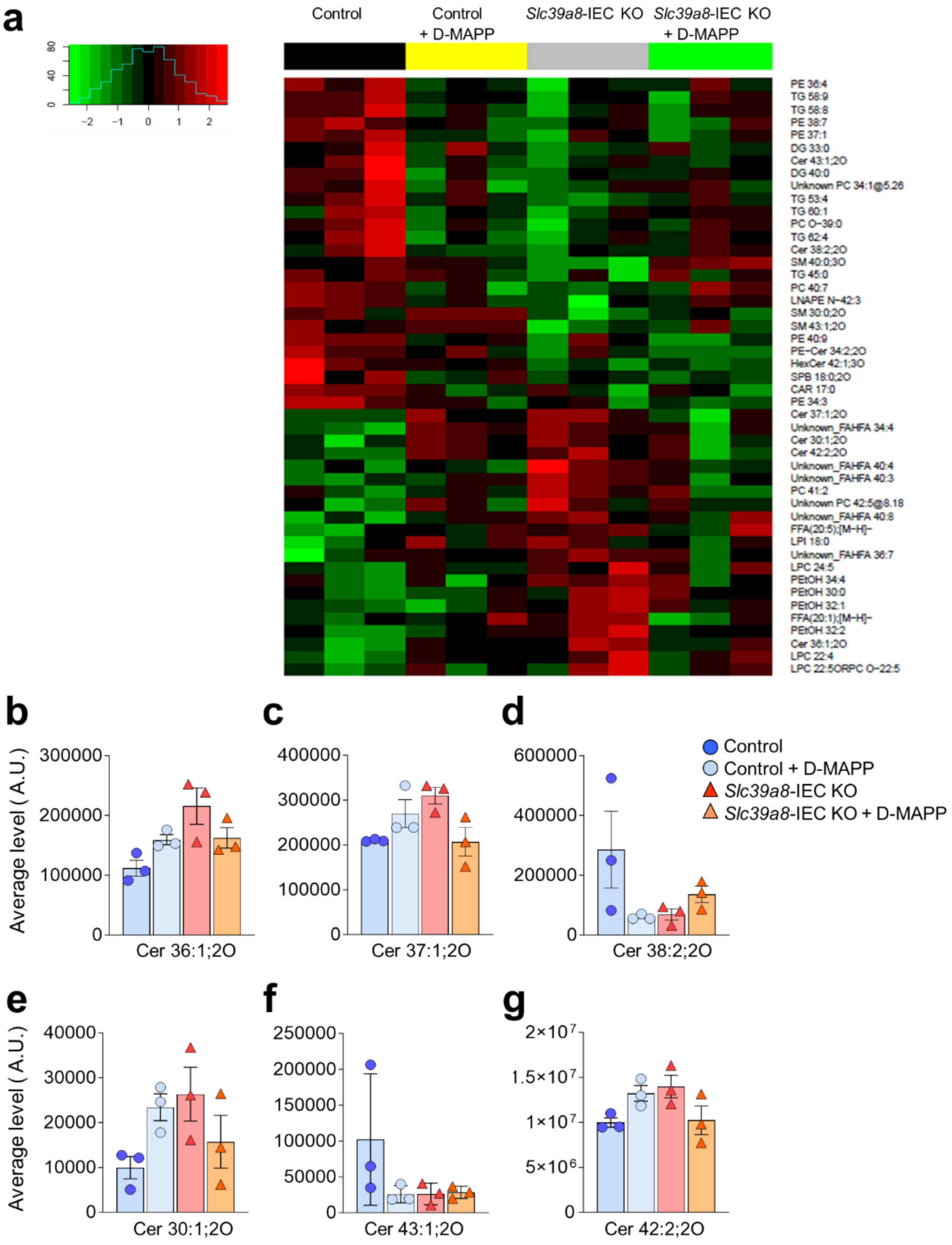

Supplementary Fig. 7. Altered lipidome in the intestines of control and *Slc39a8*-IEC KO mice with and without the ACER1 inhibitor D-*e*-MAPP. (**a**) Heat map showing significantly altered lipids in the intestines of control and *Slc39a8*-IEC KO mice with and without the ACER1 inhibitor (1S,2R)-d-erythro-2-(N-myristoylamino)-1-phenyl-1-propanol (D-*e*-MAPP). Shades of red and green represent upregulated and downregulated lipids, respectively (see color key). Lipid classes include PE: phosphatidylethanolamine, TG: triglycerides, DG: diglycerides, CE: ceramides, PC: phosphatidylcholine, SM: sphingomyelins, LNAPE: N-acyl-lysophosphatidylethanolamine, PE-Cer: phosphatidylethanolamine ceramides, HexCer: hexosylceramides, SPB: sphinganine, FFA: free fatty acids, LPI: lysophosphatidylinositol, LPC: lysophophatidylcholine, and PEtOH: phosphatidylethanol (n = 3 per group). (**b-g**) Average levels of ceramide species Cer 36:1;2O, Cer 37:1;2O, Cer 38:2;2O, Cer 30:1;2O, Cer 43:1;2O, and Cer 42:2;2O. Data are presented as individual values and represent the mean ± SEM. The *p*-values were determined by one-way ANOVA with Bonferroni's multiple comparisons test for **b-g**. Source data are provided as a Source Data file.

Supplementary Figure 8

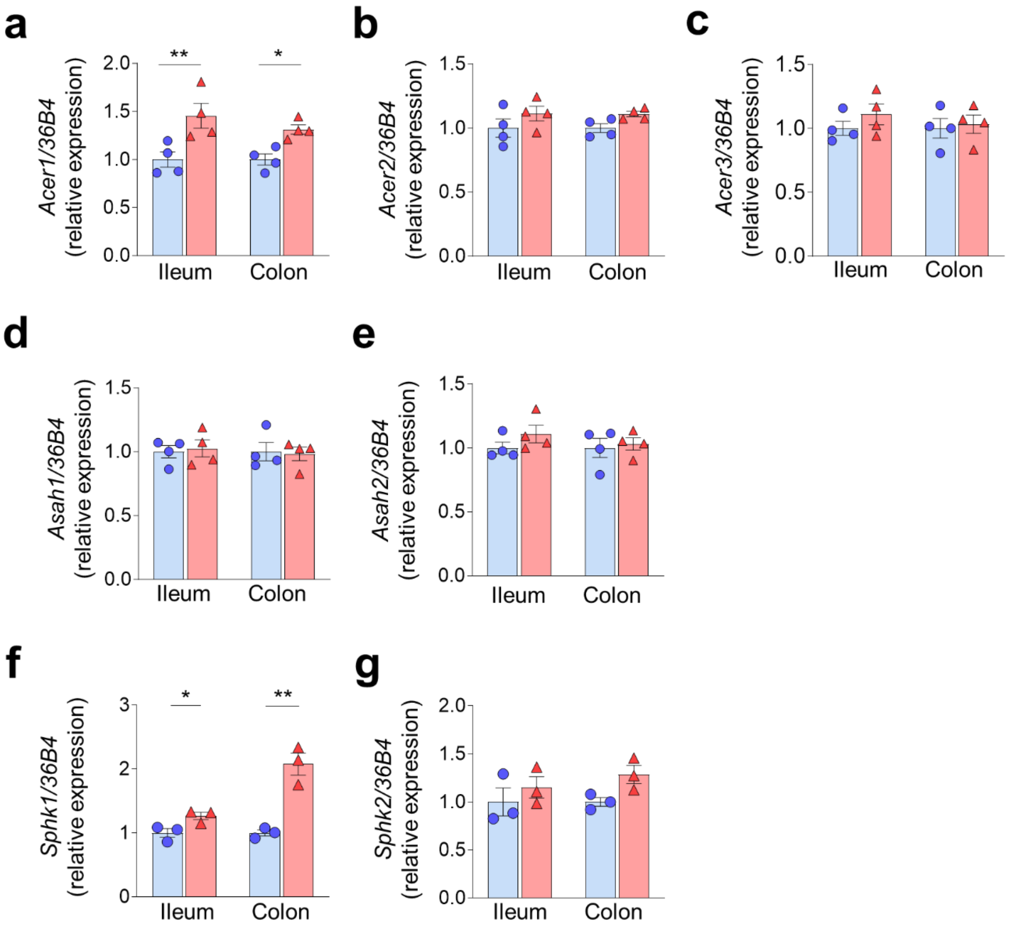

Supplementary Fig. 8. qPCR analysis of ceramidases and sphingosine kinases. (**a, b, c**) alkaline ceramidases 1, 2, and 3 (*Acer1*, *Acer2*, and *Acer3*), (**d**) acid ceramidases (*Asah1*), (**e**) neutral ceramidase (*Asah2*) in the ileal and colonic mucosa in control and *Slc39a8*-IEC KO mice (*n* = 4 per group). (**f, g**) sphingosine kinases 1 and 2 (*Sphk1* and *Sphk2*) in the ileal and colonic mucosa in control and *Slc39a8*-IEC KO mice (*n* = 3 per group). Data are presented as individual values and represent the mean ± SEM. The *p*-values were determined by unpaired two-tailed Student's *t*-test for **a-g**. Source data are provided as a Source Data file.

Supplementary Figure 9

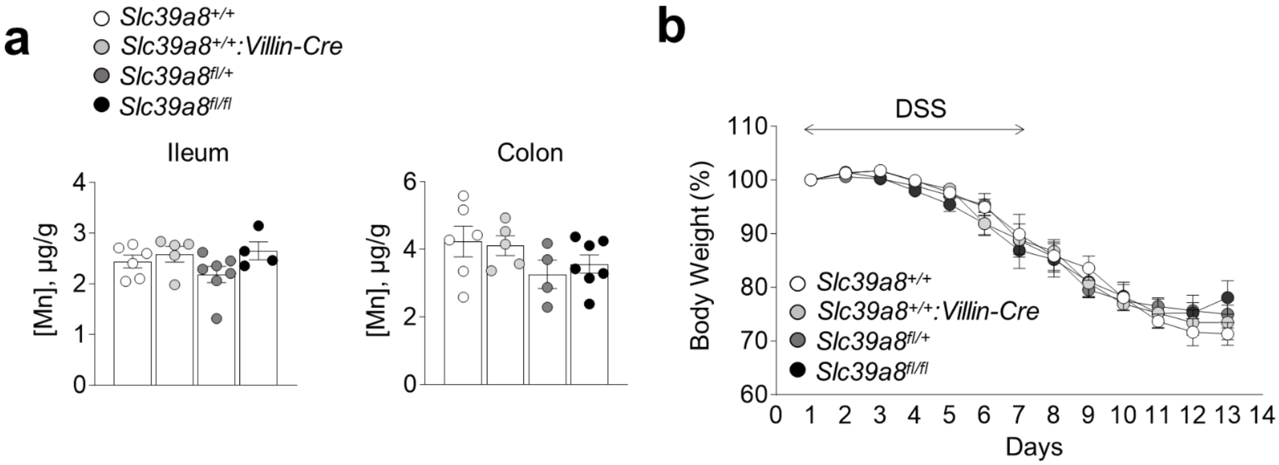

Supplementary Fig. 9. Effects of various *Slc39a8* genotypes and Cre transgenes on Mn levels and DSS sensitivity. (**a**) Manganese levels in ileum and colon in 6- to 8-week-old *Slc39a8*$^{+/+}$, *Slc39a8*$^{+/+}$*Vilin-Cre, Slc39a8*$^{fl/+}$, and *Slc39a8*$^{fl/fl}$ mice. For ileum samples, *Slc39a8*$^{+/+}$, n=6; *Slc39a8*$^{+/+}$*:Villin-Cre*, n=5; *Slc39a8*$^{fl/+}$, n=7; *Slc39a8*$^{fl/fl}$, n=4 per group. For colon samples, *Slc39a8*$^{+/+}$, n=6; *Slc39a8*$^{+/+}$*:Villin-Cre*, n=5; *Slc39a8*$^{fl/+}$, n=4, *Slc39a8*$^{fl/fl}$, n=7 per group. (**b**) DSS sensitivity in 6-8 weeks-old *Slc39a8*$^{+/+}$, *Slc39a8*$^{+/+}$*Vilin-Cre, Slc39a8*$^{fl/+}$, and *Slc39a8*$^{fl/fl}$ mice (*Slc39a8*$^{+/+}$, n=3; *Slc39a8*$^{+/+}$*:Villin-Cre*, n=3; *Slc39a8*$^{fl/+}$, n=4; *Slc39a8*$^{fl/fl}$, n=4 per group). Data are presented as individual values and represent the mean ± SEM. The *p*-values were determined by one-way ANOVA with Bonferroni's multiple comparisons test for **a** and two-way ANOVA with Bonferroni's multiple comparisons test for **b**. Source data are provided as a Source Data file.

Supplementary Figure 10

a

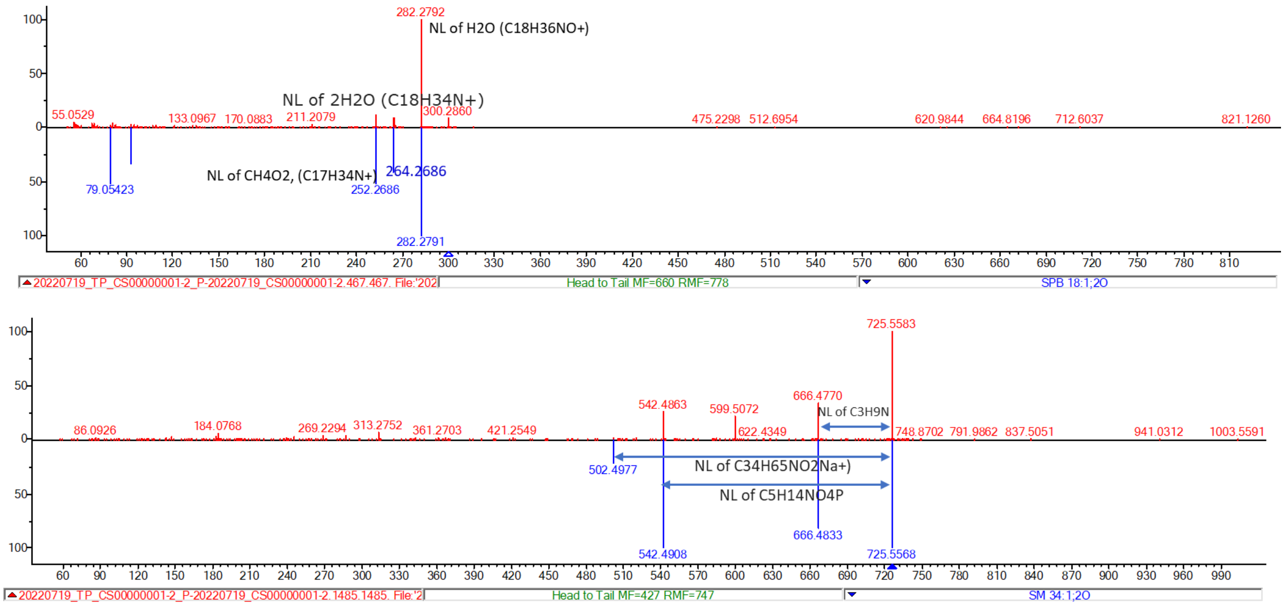

b

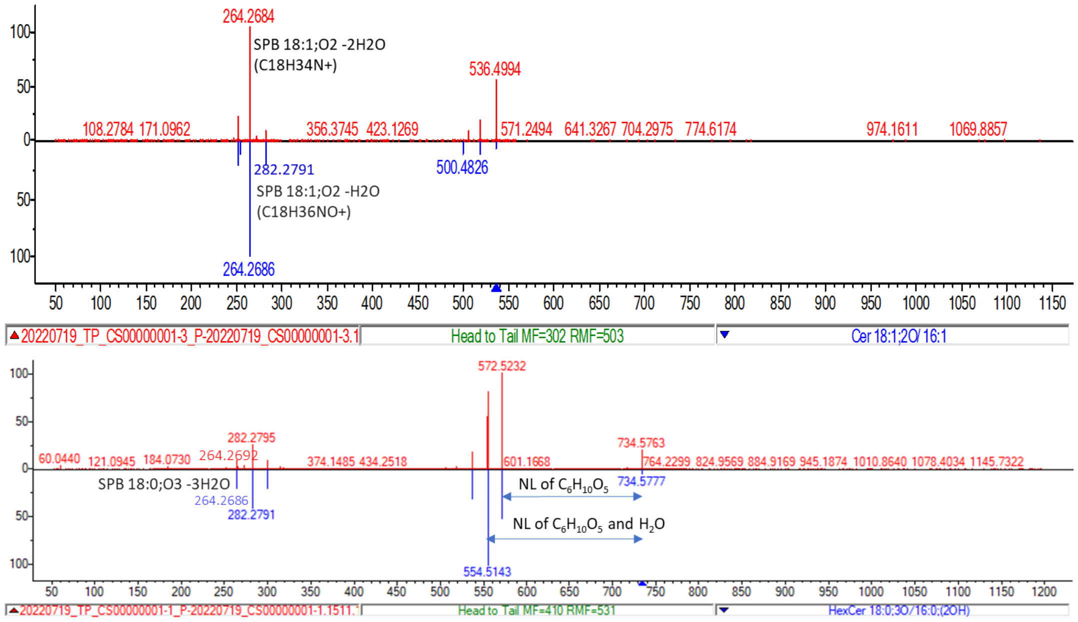

c

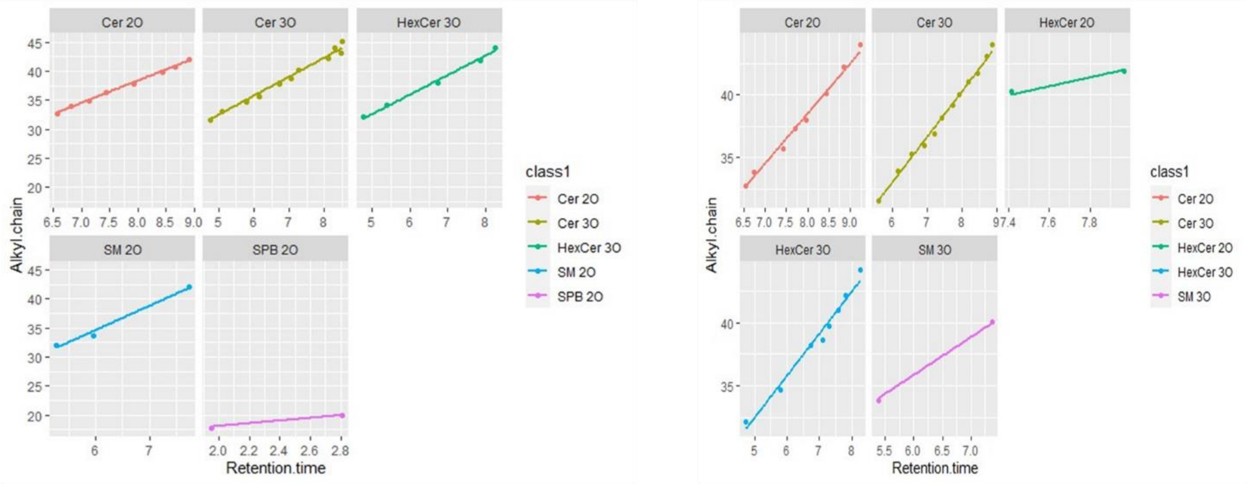

d

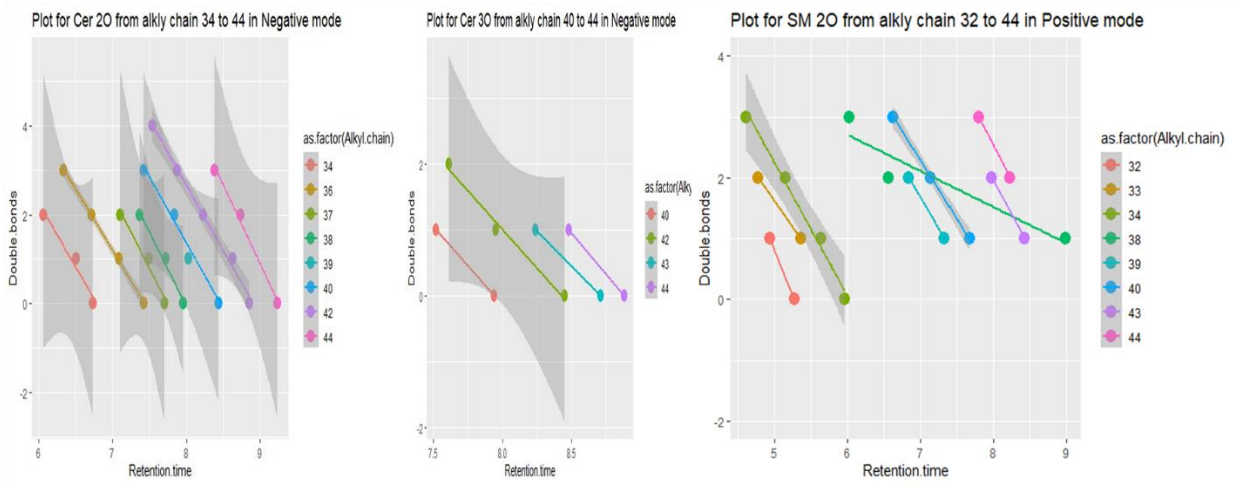

Supplementary Fig. 10. Spectral matches and Validation of sphingolipid species. (**a**) In-silico library can be used for assignment of MS/MS spectra from ABSciex 5600 Triple TOF Platform for SPB 18:1;2O $[M+H]^+$ (top) and SM 34:1;2O$[M+Na]^+$ (bottom). Experimental spectra are depicted on top (red) and in-silico MS/MS spectra are shown on the bottom (blue). (**b**) In-silico library can be used for assignment of MS/MS spectra from ABSciex 5600 Triple TOF Platform for Cer 18:1;2O/16:1 [M+H]+ (top) and HexCer 18:0;3O/16:0;(2OH) [M+H]+ (bottom). Experimental spectra are depicted on top (red) and in-silico MS/MS spectra are shown on the bottom (blue). (**c**) RT increases with increasing alkyl chain in sphingolipids. (**d**) RT decreases with increasing the number of double bonds in sphingolipids.

Supplemental Table 1. The four most dysregulated genes in *Slc39a8*-IEC KO intestines.

Genes	Gene Description	*P*adj	Known Function
**Downregulated**			
Slc39a8	Solute Carrier Family 39 Member 8	1.25×10^{-5}	Cellular import of divalent metal ions[1, 2, 3, 4]
**Upregulated**			
Ighv1-55 (ENSMUSG00000095589)	Immunoglobulin Heavy Variable 1-55	3.40×10^{-4}	Unknown function
Entpd4b	Ectonucleoside Triphosphate Diphosphohydrolase 4	2.56×10^{-2}	Hydrolysis of nucleotide diphosphates and triphosphates[5]
Acer1	Alkaline Ceramidase 1	4.65×10^{-2}	Hydrolysis of very long chain ceramides to generate sphingosine[6, 7]

The *p*-values were determined by Wald test implemented in DESeq2 R package.

Supplemental Table 2. Primer sequences used in this study.

Primer name	Sequence
Slc39a8	CTA AGA AAG CAC AAC GCA AAG CC
	CCA ATA GCG AGT CCC ACG AAA TAA G
Cldn2	TTT TCC AAG GGC CTC TGG AT
	AAG ACT CCA CCC ACT ACA GC
Cldn3	AAA TGT ACG ACT CGC TGC TG
	CGA TGG TGA TCT TGG CCT TG
Cldn4	CAT CAG CAT CAT CGT GGG TG
	TAG GGT TGT AGA AGT CGC GG
Cldn5	CGT TGT CCG CGA GTT CTA TG
	CCT CCC GCC CTT AGA CAT AG
Cldn7	GCC TTG GTA GCA TGT TCC TG
	TTT GCT TTC ACT GCC TGG AC
Ocl	CTA CGG AGG TGG CTA TGG AG
	AGC GCT GAC TAT GAT CAC GA
Zo1	ACC CGA AAC TGA TGC TGT GGA TAG
	AAA TGG CCG GGC AGA ACT TGT GTA
Zo2	GTT TGG CAG CTT GAA GGA CA
	CTC ATT GTC AGT GTA GGC GC
Il1b	GCA GCA GCA CAT CAA CAA G
	CAC GGG AAA GAC ACA GGT AG
Il6	AGA CAA AGC CAG AGT CCT TCA GA
	GCC ACT CCT TCT GTG ACT CCA
Tnfa	GCC ACC ACG CTC TTC TGT CT
	CAG CTG CTC CTC CAC TTG GT
Cxcl1	CCG AAG TCA TAG CCA CAC TCA A
	GCA GTC TGT CTT CTT TCT CCG TTA C
Ccl2	GAA GGA ATG GGT CCA GAC AT
	ACG GGT CAA CTT CAC ATT CA
Acer1	ATG CTC ATA GGT CTG TTC TC
	AGT GGT TAT AGT TAC CAG GC
Acer2	GTG TGG CAT ATT CTC ATC TG
	TAA GGG ACA CCA ATA AAA GC
Acer3	GTG TGG CAT ATT CTC ATC TG
	TAA GGG ACA CCA ATA AAA GC
Asah1	TTC TCA CCT GGG TCC TAG CC
	TAT GGT GTG CCA GGG AAC TG
Asah2	AGA GAG AGC AAG GTA TTC TTC
	ACT ATT CAC AAA GTG GTT GC
36B4	TCA TCC AGC AGG TGT TTG AC
	TAC CCG ATC TGC AGA CAC AC

Supplementary reference

1. NA B, M K, Y M, M M, K T, T S. Mycobacterium bovis BCG cell wall and lipopolysaccharide induce a novel gene, BIGM103, encoding a 7-TM protein: identification of a new protein family having Zn-transporter and Zn-metalloprotease signatures. *Genomics* **80**, (2002).

2. TP D*, et al.* Identification of mouse SLC39A8 as the transporter responsible for cadmium-induced toxicity in the testis. *Proceedings of the National Academy of Sciences of the United States of America* **102**, (2005).

3. L H*, et al.* ZIP8, member of the solute-carrier-39 (SLC39) metal-transporter family: characterization of transporter properties. *Molecular pharmacology* **70**, (2006).

4. Wang CY*, et al.* ZIP8 is an iron and zinc transporter whose cell-surface expression is up-regulated by cellular iron loading. *J Biol Chem* **287**, 34032-34043 (2012).

5. Biederbick A, Rose S, Elsässer HP. A human intracellular apyrase-like protein, LALP70, localizes to lysosomal/autophagic vacuoles. *J Cell Sci* **112 (Pt 15)**, 2473-2484 (1999).

6. Houben E*, et al.* Differentiation-associated expression of ceramidase isoforms in cultured keratinocytes and epidermis. *J Lipid Res* **47**, 1063-1070 (2006).

7. Sun W*, et al.* Upregulation of the human alkaline ceramidase 1 and acid ceramidase mediates calcium-induced differentiation of epidermal keratinocytes. *J Invest Dermatol* **128**, 389-397 (2008).
