# Supplementary material for: The manganese transporter SLC39A8 links alkaline ceramidase 1 to inflammatory bowel disease
Source: Nat Commun. 2024 Jun 5;15:4775. doi: 10.1038/s41467-024-49049-8 (PMC11153611; doi:10.1038/s41467-024-49049-8)
Supplement: Supplementary file 3 — Reporting Summary [file 41467_2024_49049_MOESM3_ESM.pdf]

nature portfolio

Corresponding author(s): Young Ah Seo

Last updated by author(s): Apr 4, 2024

Reporting Summary

Nature Portfolio wishes to improve the reproducibility of the work that we publish. This form provides structure for consistency and transparency in reporting. For further information on Nature Portfolio policies, see our Editorial Policies and the Editorial Policy Checklist.

Statistics

For all statistical analyses, confirm that the following items are present in the figure legend, table legend, main text, or Methods section.

n/a	Confirmed	
[ ]	[x]	The exact sample size (*n*) for each experimental group/condition, given as a discrete number and unit of measurement
[ ]	[x]	A statement on whether measurements were taken from distinct samples or whether the same sample was measured repeatedly
[ ]	[x]	The statistical test(s) used AND whether they are one- or two-sided<br>*Only common tests should be described solely by name; describe more complex techniques in the Methods section.*
[x]	[ ]	A description of all covariates tested
[ ]	[x]	A description of any assumptions or corrections, such as tests of normality and adjustment for multiple comparisons
[ ]	[x]	A full description of the statistical parameters including central tendency (e.g. means) or other basic estimates (e.g. regression coefficient) AND variation (e.g. standard deviation) or associated estimates of uncertainty (e.g. confidence intervals)
[ ]	[x]	For null hypothesis testing, the test statistic (e.g. *F*, *t*, *r*) with confidence intervals, effect sizes, degrees of freedom and *P* value noted<br>*Give P values as exact values whenever suitable.*
[x]	[ ]	For Bayesian analysis, information on the choice of priors and Markov chain Monte Carlo settings
[x]	[ ]	For hierarchical and complex designs, identification of the appropriate level for tests and full reporting of outcomes
[x]	[ ]	Estimates of effect sizes (e.g. Cohen's *d*, Pearson's *r*), indicating how they were calculated

Our web collection on statistics for biologists contains articles on many of the points above.

Software and code

Policy information about availability of computer code

Data collection	RNA-seq libraries were sequenced on the Illumina NovaSeq 6000 platform,w ith paired-end 150 base pair reads,a ccording to standard procedures.
Data analysis	RNA-seq reads were mapped to the mml0 mouse genome (Gencode) using STAR (v2.5.3a),w here only uniquely mapped reads were used for downstream analyses. Duplicates were removed using UMI -tools (v0.5.4),a nd a counts file was generated using FeatureCounts (Su bread vl.5.0). BAM files were converted to bigwigs using deeptools (v3.1.3). Differentially expressed (DE) genes were called using DESeq2 (vl.14.1). We used p < 0.01 to identify DE genes. Data analyses were performed with RStudio (vl.0.136) or GraphPad Prism (v7.00 or 8.02) for Mac (GrapPad Software,L a Jolla California USA,w ww.graphpad.com). Fold change heatmaps was created using shinyheatmap on the web.

For manuscripts utilizing custom algorithms or software that are central to the research but not yet described in published literature, software must be made available to editors and reviewers. We strongly encourage code deposition in a community repository (e.g. GitHub). See the Nature Portfolio guidelines for submitting code & software for further information.

Data

Policy information about availability of data

All manuscripts must include a data availability statement. This statement should provide the following information, where applicable:

- Accession codes, unique identifiers, or web links for publicly available datasets
- A description of any restrictions on data availability
- For clinical datasets or third party data, please ensure that the statement adheres to our policy

All data generated or analyzed during this study are included in this published article (and its supplementary information files). All the datasets in this study are existing published and are available via the NCBI website, including Gene Expression Omnibus (GSE) accession number: GSE192695. Lipidomics data have been deposited at https://github.com/SeoResearchLab/IECKO2023. Source data are provided with this paper.

Research involving human participants, their data, or biological material

Policy information about studies with human participants or human data. See also policy information about sex, gender (identity/presentation), and sexual orientation and race, ethnicity and racism.

Reporting on sex and gender	N/A
Reporting on race, ethnicity, or other socially relevant groupings	N/A
Population characteristics	N/A
Recruitment	N/A
Ethics oversight	N/A

Note that full information on the approval of the study protocol must also be provided in the manuscript.

Field-specific reporting

Please select the one below that is the best fit for your research. If you are not sure, read the appropriate sections before making your selection.

☒ Life sciences ☐ Behavioural & social sciences ☐ Ecological, evolutionary & environmental sciences

For a reference copy of the document with all sections, see nature.com/documents/nr-reporting-summary-flat.pdf

Life sciences study design

All studies must disclose on these points even when the disclosure is negative.

Sample size	Sample size was chosen to ensure reproducibility of the experiments in accordance with the replacement, reduction, and refinement principles of animal ethics regulation. Sample size in this study were based on previous studies with similar experiments, e.g. PMID: 6877324 (Mercadante et al., 2019 JCI), e.g. PMID: 29437953 (Jenkitkasemwong, et al. 2018 PNAS).
Data exclusions	Outliers were identified using the Graph Pad ROUT (robust regression and outlier removal) method (Q = 1%).
Replication	We previously did a number of studies on the impact of manganese on experimental colitis model, metal analysis, and histological analysis (Choi et al., 2020) and RNA-seq (Seo et al., 2021). All were consistent with previous findings. All experimental findings were reliably reproduced. All in vitro and in vivo experiments were carried out with at least 3 biological replicates for each experimental group.
Randomization	All samples were randomly allocated into experimental groups.
Blinding	In animal experiments and cell experiments, data were collected in a blind fashion, where mice or cells were coded and genotypes only revealed after testing was complete.

Reporting for specific materials, systems and methods

We require information from authors about some types of materials, experimental systems and methods used in many studies. Here, indicate whether each material, system or method listed is relevant to your study. If you are not sure if a list item applies to your research, read the appropriate section before selecting a response.

Materials & experimental systems

n/a	Involved in the study	
[ ]	[x]	Antibodies
[x]	[ ]	Eukaryotic cell lines
[x]	[ ]	Palaeontology and archaeology
[ ]	[x]	Animals and other organisms
[x]	[ ]	Clinical data
[x]	[ ]	Dual use research of concern
[x]	[ ]	Plants

Methods

n/a	Involved in the study	
[x]	[ ]	ChIP-seq
[x]	[ ]	Flow cytometry
[x]	[ ]	MRI-based neuroimaging

Antibodies

Antibodies used

Slc39a8 Polyclonal antibody (Proteintech, 20459-1-AP), IF: 1:50
ZO-1 Monoclonal antibody (Santa Cruz, sc-33725), IF: 1:50, Western blot: 1:500
ZO-2 Monoclonal antibody (Santa Cruz; sc-515115), IF: 1:50, Western blot: 1:500
Claudin-2 Polyclonal antibody (Thermo Fisher, # 51-6100), IF: 1:100, Western blot: 1:1000
Claudin-3 Polyclonal antibody (Thermo Fisher, # 34-1700), IF: 1:100, Western blot: 1:1000
Claudin-5 Monoclonal antibody (Thermo Fisher, # 35-2500), IF: 1:50, Western blot: 1:1000
Claudin-7 Polyclonal antibody (Thermo Fisher, # 34-9100), IF: 1:50, Western blot: 1:1000
Occludin Monoclonal antibody (Santa Cruz, sc-133256), IF: 1:50, Western blot: 1:1000
Cytokeratin 8/18 Monoclonal antibody (Abcam, ab53280), Western blot: 1:50000
ASAH3 polyclonal antibody (Thermo Fisher, # PA5-75603), Western blot: 1:500
Actin Monoclonal (Proteintech, 66009-1-lg), Western blot: 1:3000
Donkey anti-rat IgG H&L (Alexa Fluor 488) (Thermo Fisher, #A48269), IF: 1:500
Donkey anti-mouse IgG H&L (Alexa Fluor 488) (Thermo Fisher, # A32766), IF: 1:1000
Donkey anti-rabbit IgG H&L (Alexa Fluor 488) (Thermo Fisher, # A32790), IF: 1:000
Goat anti-mouse IgG H&L (Alexa 488 Fisher A11001), IF: 1:1500
Goat anti-rabbit IgG H&L (Alexa 568 Fisher A11036), IF: 1:1500
IRDye 680RD Goat anti-rat IgG Secondary antibody (Liborbio, #926-69076), Western blot: 1:20000
IRDye 800CW Donkey anti-mouse IgG Secondary antibody (Liborbio, #925-32212), Western blot: 1:20000
IRDye 680LT Donkey anti-rabbit IgG Secondary antibody (Liborbio, #926-69021), Western blot: 1:15000

Validation

All antibodies were commercially available and are validated by the vendor on their official website.
Slc39a8 Polyclonal antibody (Proteintech, 20459-1-AP), IF: 1:50
https://www.thermofisher.com/antibody/product/ZIP8-Antibody-Polyclonal/20459-1-AP
ZO-1 Monoclonal antibody (Santa Cruz, sc-33725), IF: 1:50, Western blot: 1:500
https://www.scbt.com/p/zo-1-antibody-r40-76
ZO-2 Monoclonal antibody (Santa Cruz; sc-515115), IF: 1:50, Western blot: 1:500
https://www.scbt.com/p/zo-2-antibody-e
Claudin-2 Polyclonal antibody (Thermo Fisher, # 51-6100), IF: 1:100, Western blot: 1:1000
https://www.thermofisher.com/antibody/product/Claudin-2-Antibody-clone-MH44-Polyclonal/51-6100
Claudin-3 Polyclonal antibody (Thermo Fisher, # 34-1700), IF: 1:100, Western blot: 1:1000
https://www.thermofisher.com/antibody/product/Claudin-3-Antibody-Polyclonal/34-1700
Claudin-5 Monoclonal antibody (Thermo Fisher, # 35-2500), IF: 1:50, Western blot: 1:1000
https://www.thermofisher.com/antibody/product/Claudin-5-Antibody-clone-4C3C2-Monoclonal/35-2500
Claudin-7 Polyclonal antibody (Thermo Fisher, # 34-9100), IF: 1:50, Western blot: 1:1000
https://www.thermofisher.com/antibody/product/Claudin-7-Antibody-Polyclonal/34-9100
Occludin Monoclonal antibody (Santa Cruz, sc-133256), IF: 1:50, Western blot: 1:1000
https://www.scbt.com/p/occludin-antibody-e-5
Cytokeratin 8/18 Monoclonal antibody (Abcam, ab53280), Western blot: 1:50000
https://www.abcam.com/products/primary-antibodies/cytokeratin-8-antibody-ep1628y-cytoskeleton-marker-ab53280.html
ASAH3 polyclonal antibody (Thermo Fisher, # PA5-75603), Western blot: 1:500
https://www.thermofisher.com/antibody/product/ASAH3-Antibody-Polyclonal/PA5-75603
Actin Monoclonal (Proteintech, 66009-1-lg), Western blot: 1:3000
https://www.ptglab.com/products/Pan-Actin-Antibody-66009-1-Ig.htm
Donkey anti-rat IgG H&L (Alexa Fluor 488) (Thermo Fisher, #A48269), IF: 1:500
https://www.thermofisher.com/antibody/product/Donkey-anti-Rat-IgG-H-L-Highly-Cross-Adsorbed-Secondary-Antibody-Polyclonal/A48269
Donkey anti-mouse IgG H&L (Alexa Fluor 488) (Thermo Fisher, # A32766), IF: 1:1000
https://www.thermofisher.com/antibody/product/Donkey-anti-Mouse-IgG-H-L-Highly-Cross-Adsorbed-Secondary-Antibody-Polyclonal/A32766
Donkey anti-rabbit IgG H&L (Alexa Fluor 488) (Thermo Fisher, # A32790), IF: 1:000
https://www.thermofisher.com/antibody/product/Donkey-anti-Rabbit-IgG-H-L-Highly-Cross-Adsorbed-Secondary-Antibody-Polyclonal/A32790

Goat anti-mouse IgG H&L (Alexa 488 Fisher A11001), IF: 1:1500
https://www.thermofisher.com/antibody/product/Goat-anti-Mouse-IgG-H-L-Cross-Adsorbed-Secondary-Antibody-Polyclonal/A-11001
Goat anti-rabbit IgG H&L (Alexa 568 Fisher A11036), IF: 1:1500
https://www.thermofisher.com/antibody/product/Goat-anti-Rabbit-IgG-H-L-Highly-Cross-Adsorbed-Secondary-Antibody-Polyclonal/A-11036
IRDye 680RD Goat anti-rat IgG Secondary antibody (Liborbio, #926-68076), Western blot: 1:20000
https://www.licor.com/bio/reagents/irdye-680rd-goat-anti-rat-igg-secondary-antibody
IRDye 800CW Donkey anti-mouse IgG Secondary antibody (Liborbio, #925-32212), Western blot: 1:20000
https://www.licor.com/bio/reagents/irdye-800CW-donkey-anti-mouse-igg-secondary-antibody
IRDye 680LT Donkey anti-rabbit IgG Secondary antibody (Liborbio, #926-69023), Western blot: 1:15000
https://www.licor.com/bio/reagents/irdye-680lt-donkey-anti-rabbit-igg-secondary-antibody

Animals and other research organisms

Policy information about studies involving animals; ARRIVE guidelines recommended for reporting animal research, and Sex and Gender in Research

Laboratory animals

Species: Mus musculus.
SEX: female/male.
Information of sex and age was indicated in the figure legends.
Strains: all mice were in C57BL/6J background.
All mice were housed in a pathogen-free animal facility at 22 °C with 40–60% humidity on a 12-h light/dark cycle, and provided the standard rodent diet of our institution (PicoLab Laboratory Rodent Diet 5L0D, LabDiet; 70 ppm Mn) and water ad libitum, unless otherwise indicated.

Wild animals

NA

Reporting on sex

Both males and females were used.

Field-collected samples

NA

Ethics oversight

All animal studies were approved by the Institutional Animal Care and Use Committee of the University of Michigan (PRO00008963).

Note that full information on the approval of the study protocol must also be provided in the manuscript.

Plants

Seed stocks

NA

Novel plant genotypes

NA

Authentication

NA
